# Editorial: CNS myeloid cell function in health and disease

**DOI:** 10.3389/fimmu.2024.1459138

**Published:** 2024-07-17

**Authors:** Christian W. Keller, Sarah Mundt, Benjamin M. Segal

**Affiliations:** ^1^ Department of Neurology with Institute of Translational Neurology, University Hospital Münster, Münster, Germany; ^2^ Institute of Experimental Immunology, University of Zurich, Zurich, Switzerland; ^3^ Department of Neurology, The Ohio State University Wexner Medical Center, Columbus, OH, United States; ^4^ The Neuroscience Research Institute, The Ohio State University, Columbus, OH, United States

**Keywords:** CNS, myeloid cells, neuroinflammation, neurodegeneration, microglia, innate immunity, antigen presentation, neutrophils

The healthy central nervous system (CNS) harbors an ontogenetically heterogeneous pool of myeloid cells that facilitates the maintenance of tissue integrity and function during development, adulthood and senescence (1, 2). In recent years, the advent of novel single-cell-based technologies such as high-dimensional mass spectrometry or RNA sequencing (scRNA-seq) in combination with unbiased computational tools, has allowed for the identification of various CNS myeloid subsets with distinct functions during homeostasis and disease (3, 4). It is now evident that the CNS accommodates a complex phenotypical and functional heterogeneity within this innate immune cell compartment even under steady-state conditions, that is only surpassed by the reactive cellular diversity during pathology. At large, CNS-resident myeloid cells can be discerned into parenchymal cells (such as microglia) and the non-parenchymal compartment including dendritic cells and macrophages, all of which occupy uniquely assigned tasks within the continuous maintenance of tissue homeostasis and immune surveillance (5–7).

Ample studies have revealed involvement of discrete myeloid subsets in both the emergence and resolution of acquired CNS pathologies including inflammatory, degenerative and vascular conditions. Especially during disease, CNS resident myeloid cells are complemented by the migration and recruitment of blood-borne hematopoietic myeloid cells including neutrophils, adding further to the cellular complexity in the CNS and its interfaces. Myeloid cells in the CNS mediate synapse development and plasticity, scavenge material, survey and patrol against invading pathogens, facilitate tissue repair and present antigens (8, 9). While in recent years a tremendous body of research has shed light on the diversity of CNS resident/infiltrating myeloid cells and helped in the phenotypical characterization of these subsets, only little is known regarding strategies to harness this knowledge to mitigate disease. A better understanding of myeloid cell function in the CNS is warranted in order to develop novel myeloid cell-targeting therapies in the management of CNS diseases (10). This Research Topic aims to bring forward a granular understanding of CNS myeloid cell function during homeostasis and pathology.

Helbing et al. investigate the effects of lipopolysaccharide (LPS) preconditioning on microglial responses to ischemic brain injury in mice. Their research demonstrates that LPS preconditioning induces neuroprotection, evidenced by reduced brain infarction volume. Using mass spectrometry, the study identifies specific proteomic changes in microglia post-ischemia, highlighting the role of type I interferons and the STAT1/2 pathway in this protective mechanism. These findings underscore the importance of innate immune pathways in preconditioning and suggests potential therapeutic targets for ischemic stroke through modulation of microglial activity.

Spiteri et al. study the effects of PLX5622, a colony stimulating factor 1 receptor (CSF-1R) inhibitor, in a murine model of West Nile virus encephalitis (WNE). Their research reveals that PLX5622, commonly used to deplete microglia, also significantly reduces mature Ly6C^hi^ monocytes in the bone marrow, thereby impairing their recruitment to the CNS. This reduction leads to decreased neuroinflammation and improved clinical outcomes. The cessation of PLX5622 treatment reverses these protective effects, highlighting its role in inhibiting CSF-1R signaling rather than other kinases. The study emphasizes the broader implications of PLX5622 beyond microglia depletion, demonstrating its potential therapeutic benefits in mitigating tissue damage during CNS viral infections and monocyte-mediated diseases, while advising caution in interpreting results assuming a microglia-specific mechanism of action.

Recent studies revealed significant heterogeneity among neutrophils. Jerome et al. characterized a novel subset of neutrophils with neuroregenerative properties following administration of zymosan, a fungal cell wall extract. These immature Ly6G^lo^ neutrophils, which appear three days post-injection, express markers of alternative activation and possess neuroprotective abilities, enhancing neuron survival and axon regeneration *in vivo*. In contrast, conventional, mature Ly6G^hi^ neutrophils, present four hours post-injection, lack these regenerative properties. The study utilized single-cell RNAseq and proteomics to demonstrate distinct gene and protein expression profiles between the two subsets, with the three-day neutrophils upregulating genes associated with tissue development and wound healing, providing insights into potential therapeutic applications for immune driven neuroregeneration. ​

Skinner et al. investigate the role of sustained neutrophil infiltration in the CNS in a virus-induced model of multiple sclerosis (MS). Using a transgenic mouse model with inducible expression of the chemokine CXCL1, they observed increased neutrophil presence in the CNS, which correlated with heightened demyelination and clinical disease severity. Single-cell RNAseq and flow cytometry analyses revealed that these neutrophils exhibit distinct gene and protein expression profiles associated with inflammation and tissue damage. The study suggests that chronic infiltration by pro-inflammatory neutrophil subsets exacerbates white matter damage through enhanced chemotaxis and activation of effector functions, highlighting potential therapeutic targets to mitigate demyelination in MS.

Finally, Chang et al. developed a CRISPR-based genome editing platform to enhance microglial research, specifically using the human microglial cell line HMC3. By electroporating Cas9 ribonucleoproteins and synthetic DNA repair templates, they achieved efficient gene knockouts and insertions with minimal off-target effects. The study targeted genes involved in amyloid-beta (Aβ) and glioblastoma phagocytosis, demonstrating increased phagocytic activity in genetically modified HMC3 cells. This approach facilitates the functional interrogation of microglial biology and provides a robust tool for investigating microglia-related neurodegenerative diseases and brain tumors.

## Author contributions

CK: Writing – review & editing, Writing – original draft. SM: Writing – review & editing, Writing – original draft. BS: Writing – review & editing, Writing – original draft.
